# Gradient boosting-based classification of interactome hub genes in periimplantitis with periodontitis – an integrated bioinformatic approach

**DOI:** 10.3389/froh.2024.1462845

**Published:** 2024-11-26

**Authors:** Pradeep Kumar Yadalam, Sarvagya Sharma, Prabhu Manickam Natarajan, Carlos M. Ardila

**Affiliations:** ^1^ Department of Periodontics, Saveetha Dental College and Hospitals, Saveetha Institute of Medical and Technical Sciences, Saveetha University, Chennai, India; ^2^ Department of Clinical Sciences, Center of Medical and Bio-allied Health Sciences and Research, College of Dentistry, Ajman University, Ajman, United Arab Emirates; ^3^ Department of Basic Sciences, Biomedical Stomatology Research Group, School of Dentistry, University of Antioquia, Medellín, Colombia

**Keywords:** peri-implantitis, machine learning, computational biology, genes, gene regulatory networks

## Abstract

**Introduction:**

Peri-implantitis, a destructive inflammatory condition affecting the tissues surrounding dental implants, shares pathological similarities with periodontitis, a chronic inflammatory disease that impacts the supporting structures of natural teeth. This study utilizes a network-based approach to classify interactome hub genes associated with peri-implantitis and periodontitis, aiming to improve understanding of disease mechanisms and identify potential therapeutic targets.

**Methods:**

We employed gradient boosting and Weighted Gene Co-expression Network Analysis (WGCNA) to predict and classify these interactome hub genes. Gene expression data related to these diseases were sourced from the NCBI GEO dataset GSE223924, and differential gene expression analysis was conducted using the NCBI GEO R tool. Through WGCNA, we constructed a co-expression network to identify key hub genes, while gradient boosting was used to predict these hub genes.

**Results:**

Our analysis revealed a co-expression network comprising 216 genes, including prominent hub genes such as IL17RC, CCN2, BMP7, TPM1, and TIMP1, which are implicated in periodontal disease. The gradient boosting model achieved an 88.2% accuracy in classifying interactome hub genes in samples related to peri-implantitis and periodontitis.

**Discussion:**

These identified genes play roles in inflammation, osteoclast genesis, angiogenesis, and immune response regulation. This study highlights essential hub genes and molecular pathways associated with peri-implantitis and periodontitis, suggesting potential therapeutic targets for developing innovative treatment strategies.

## Introduction

Peri-implantitis is an inflammatory condition affecting the tissues surrounding dental implants, characterized by inflammation and progressive bone loss (1). The progression from mucositis to peri-implantitis is not yet fully understood, displaying an early onset and a nonlinear pattern of advancement. Clinically, peri-implantitis is marked by signs of inflammation, increased probing depths, and larger inflammatory lesions than those typically seen in periodontitis. Key risk factors for peri-implantitis include a history of chronic periodontitis and inadequate plaque control, though associations with smoking and diabetes remain inconclusive (1–3).

In cases of peri-implantitis, the mean probing depth (PD) and clinical attachment level (CAL) are significantly greater at implant sites affected by peri-implantitis compared to other sites. Moreover, the presence of peri-implantitis, implant location, and examination site have all been shown to be significantly associated with periodontal measurements at adjacent teeth (2, 3). Studies have also noted that the mean PD and CAL differ notably at sites near implants with peri-implantitis compared to distant sites (4–6). The presence of peri-implantitis and the location of the tooth were significantly associated with PD and CAL values. Furthermore, the examination sites (proximal to or away from the affected tooth) were significantly associated with CAL and gingival recession (GR) (5, 6).

Peri-implantitis and periodontitis are inflammatory conditions (1, 7). Both diseases share etiological factors, such as bacterial colonization, host immune response, genetic predisposition, and environmental factors (6–8). Common pathogens associated with periodontitis include *Porphyromonas gingivalis*, *Treponema denticola*, and *Tannerella forsythia*. Research shows that periodontitis increases the risk of developing peri-implantitis, with patients with a history of periodontitis showing a higher incidence of peri-implant inflammation and destruction (4, 5, 9, 10). Peri-implantitis and periodontitis involve Gram-negative bacteria, but peri-implantitis has a more aggressive inflammatory response, larger, more vascularized lesions, and higher levels of matrix metalloproteinases (MMP). The disease progresses more rapidly, leading to quicker and more severe bone loss, likely due to differences in microbial composition and host defense mechanisms. Peri-implantitis causes faster and more severe bone loss than periodontal disease due to a nonlinear progression of bone destruction influenced by microorganisms, host defense mechanisms, and the absence of a periodontal ligament (11).

The pathogenesis involves the host immune response, bone homeostasis, and genetic factors (1). A comprehensive review of clinical research indicated that periodontitis increases the likelihood of peri-implantitis (7). Peri-implantitis is an inflammatory condition characterized by the inflammation of the tissues surrounding dental implants, leading to bone loss and potential implant failure. Understanding the gene expression profiles associated with peri-implantitis can help identify biomarkers for diagnosis, pathogenesis, and potential therapeutic targets (11). Key aspects of gene expression analysis include the inflammatory response, bone remodeling and resorption, matrix metalloproteinases (MMPs), microbiome influence, immune response genes, and gene expression technologies. Peri-implantitis patients often have elevated inflammatory response genes and bone remodeling and resorption genes, indicating an active inflammatory process, potential tissue destruction, and impaired healing. Peri-implantitis is influenced by the microbiome, with immune response genes upregulated or downregulated (9–11). Gene expression technologies like PCR, microarray analysis, and RNA sequencing aid in understanding the disease's molecular landscape.

Peri-implantitis increases the risk of complications in implant therapy, including marginal bone loss and implant loss, and is a significant factor in implant rehabilitation rates, according to systematic reviews. Gene expression analysis showed higher levels of proinflammatory markers in peri-implantitis in periodontitis, and one previous study compared peri-implantitis and periodontitis lesions, revealing differences in tissue structure and vascular density, with conflicting data on vascular density in peri-implantitis and periodontitis tissues (8, 12, 13).

Weighted Gene Co-expression Network Analysis (WGCNA) (14–16) is a computational method that identifies genes with highly correlated expression patterns by calculating pairwise correlations. WGCNA is a powerful tool for analyzing high-dimensional data, aiding in identifying key gene interactions, biomarkers, and biological mechanisms in fields like genomics, transcriptomics, and systems biology (14, 15). It groups genes into modules based on topological overlap measures, providing insights into gene regulatory networks, potential biomarkers, and key disease drivers. The system creates a network of genes based on expression data, identifying highly correlated genes that may share biological functions or co-regulate each other (14–16).

Interactome hub genes are central hubs in molecular interaction networks, mediating interactions between molecules and pathways. They regulate multiple biological processes simultaneously, potentially influencing disease development (14, 17). Studying these hub genes helps identify therapeutic targets and develop personalized medicine approaches.

Machine learning analyses and predicts interactions between interactome hub genes using molecular interaction networks and gene expression data (18, 19). This process includes feature engineering, model selection, training, validation, and application to new data, providing insights into biological processes. A recent study developed an Artificial Neural Network model for early diagnosis of Periprosthetic Infection (PI). It identified 1,380 differentially expressed genes, highlighting neutrophil-mediated immunity and NF-kappa B signaling pathways. The model accurately diagnosed PI at an early stage using 13 hub genes (20). Another study found distinct risk groups for peri-implantitis based on immune profiles, microbial dynamics, and regenerative outcomes. Low-risk patients had higher macrophage ratios, reduced B-cell infiltration, and enriched *Fusobacterium nucleatum* and *Prevotella intermedia* (21).

Research on the interactome gene analysis of peri-implant disease is currently limited. Comprehensive studies are needed to better understand the molecular mechanisms involved and their potential connections with periodontal diseases. This knowledge could aid in the development of effective prevention and treatment strategies. Accordingly, our study aims to predict and classify interactome hub genes by using gradient boosting and WGCNA.

## Materials and methods

### Dataset preparation

The dataset utilized for this study was obtained from the NCBI Gene Expression Omnibus (GEO) repository under accession number GSE223924 (19). This dataset comprises samples from 20 participants, including 10 healthy individuals and 10 patients diagnosed with periodontitis and peri-implantitis. Tissue samples from healthy individuals and those affected by periodontitis and peri-implantitis were collected and subjected to genetic analysis.

### Differential gene expression analysis

Differential gene expression analysis was conducted using the GEO2R tool (19). It facilitates the process by locating the desired dataset, accessing it, selecting target and control samples, applying appropriate normalization techniques, and initiating the analysis. The tool performs background correction, normalization, and statistical analysis to identify differentially expressed genes. The results are tabular, with genes ranked based on their fold change or *p*-value significance. Users can further explore the results by selecting specific genes and visualizing their expression patterns through various plots available within GEO2R.

### Cytoscape – interactome hub genes

Network analysis using Cytoscape (22) and the CytoHubba plugin involves adjusting parameters, running hub gene analysis, and analyzing the results. CytoHubba generates a ranked list of hub genes based on their importance scores, which can be visualized by highlighting, adjusting node size or color, or creating subnetworks.

### Gene ontology – Enrichr

Enrichr performs enrichment analysis (23) using the Gene Ontology database. It provides tabulated results containing enriched GO terms, associated *p*-values, and gene counts. Additionally, Enrichr offers visual representations and bar plots for interpretation, aiding researchers in understanding overrepresented biological functions and identifying pathways.

### WGCNA analysis

WGCNA is a bioinformatics method employed for analyzing high-dimensional gene expression data. It aids in identifying co-expression modules of genes and uncovering gene networks correlated with specific phenotypes or conditions. The WGCNA process encompasses data preprocessing, constructing a co-expression similarity matrix, network construction, module identification, module preservation, and stability analysis, functional enrichment analysis, and module-trait association analysis. Data undergoes normalization and log transformation, with quality control steps executed to eliminate low-quality or unreliable data. The resultant matrix is transformed into an adjacency matrix, indicating the strength of gene connections. WGCNA further facilitates functional enrichment analysis to explore biological processes, pathways, or gene ontology terms linked with genes within each module (24).

### IDEP

Integrated Differential Expression And Pathway Analysis (IDEP) (24) is a user-friendly web server providing a comprehensive analysis pipeline for gene expression data, including WGCNA. To perform WGCNA analysis using IDEP, prepare your gene expression data in the appropriate format and upload it to the IDEP website. Then, select the suitable data type and normalization method, furnish experimental information, conduct quality control checks, identify differentially expressed genes, and opt for the “Weighted Gene Co-expression Network Analysis (WGCNA)” feature in the “Gene Networks” section. Adjust parameters such as soft thresholding power and minimum module size, and initiate the WGCNA analysis by clicking the “Run Analysis” button. IDEP generates a WGCNA network and identifies co-expression modules based on the specified parameters. IDEP furnishes visualizations and downloadable results post-analysis, enabling exploration of the network dendrogram, module-trait relationships, gene significance plots, and functional enrichment analysis results.

## Machine learning of hub genes of top DEGs

### Dataset preparation

The study aimed to identify the top 100 hub genes from differentially expressed genes (DEGs) to gain insights into biological processes. Data preprocessing ensured data quality, and the performance of the gradient boosting algorithm was assessed by dividing the dataset into training and test sets, ensuring sufficient data for training and evaluating model performance. The gradient boosting algorithm (25), an ensemble method, was utilized on the training dataset, leveraging multiple weak prediction models to construct a robust and accurate model for exploring gene interactions.

### Gradient boosting architecture

The Gradient Boosting model with CatBoost architecture was configured with a learning rate of 0.009, comprising 26 trees, a regularization lambda of 0.05, and a limit of 5 individual trees. This setup balances model complexity and regularization, aiming to minimize overfitting and achieve optimal performance in predicting interactome hub genes associated with peri-implantitis and periodontitis. The model is trained with 26 trees, with a higher lambda value employed to mitigate overfitting. The model's limit also ensures a maximum of 5 trees in the final ensemble.

The Gradient Boosting model with CatBoost presents several advantages over alternative models. It automatically handles categorical variables, reducing the necessity for manual preprocessing. Regularization techniques control overfitting by managing the shrinkage of individual tree weights, thereby enhancing generalization to unseen data. The adaptive learning rate, set at 0.009, diminishes overshooting and enhances convergence speed. Limiting the number of individual trees helps prevent overfitting and excessive model complexity. The architecture and parameters have been fine-tuned to predict interactome hub genes associated with peri-implantitis and periodontitis, resulting in enhanced predictive performance. Accuracy assessment was conducted using the orange machine learning tool (25).

## Results

**Figure 1:**
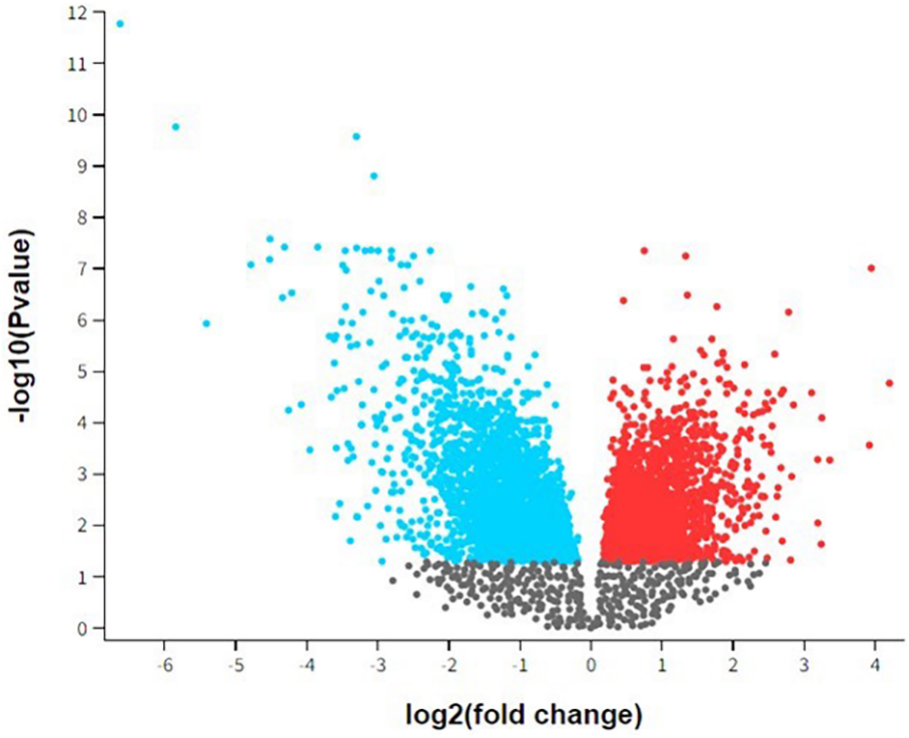
Volcano plot of top differential gene expression in peri-implantitis and periodontitis.

Figure 1 presents a volcano plot depicting the differential gene expression observed in peri-implantitis and periodontitis. This volcano plot visualizes the differential gene expression between peri-implantitis and periodontitis. Each dot represents a gene, plotted according to its statistical significance and magnitude of expression change. The *x*-axis, labeled as “log2 (fold change),” indicates the degree of expression change for each gene. Genes on the right side (positive values) are upregulated, while those on the left (negative values) are downregulated. The *y*-axis, labeled “-log10 (*p*-value),” represents the statistical significance of each gene's differential expression. Higher points indicate greater significance (lower *p*-values). In the plot, red dots represent significantly upregulated genes (*p* < 0.05 and log2 fold change > 1), while blue dots represent significantly downregulated genes (*p* < 0.05 and log2 fold change < −1). Gray dots represent genes with changes that are not statistically significant, falling below the typical significance threshold. This visualization helps identify key genes with substantial expression changes, which may play a role in the pathogenesis or progression of peri-implantitis and periodontitis, aiding in the identification of potential biomarkers or therapeutic targets.

**Figure 2:**
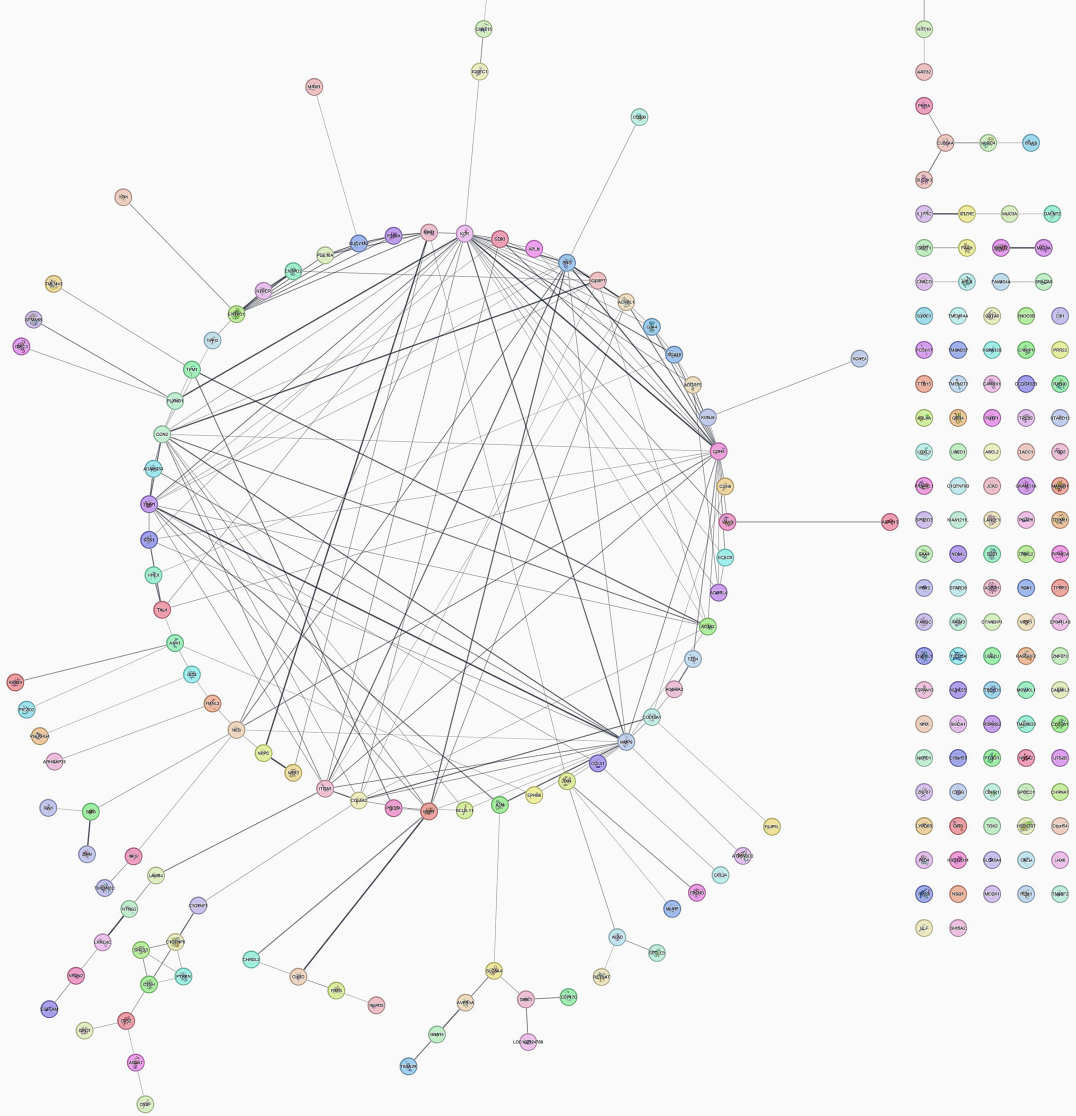
Interactome of the top 250 genes of differential gene expression.

Figure 2 showcases the interactome of the top 250 genes identified by analyzing differential gene expression. This network visualization presents a comprehensive view of the molecular interactions and relationships between these genes, shedding light on the underlying biological processes involved in peri-implantitis and periodontitis.

This network comprises 216 nodes interconnected by 206 edges, demonstrating an average of 3.712 neighboring nodes per node. The network exhibits a diameter of 12 and a radius of 6, indicating the maximum and minimum eccentricity of any node in the network. The characteristic path length of the network is calculated as 4.409, representing the average shortest path length between all pairs of nodes. Additionally, the network demonstrates a clustering coefficient of 0.228, indicating the degree to which nodes tend to cluster together. The density of the network, reflecting the ratio of the observed connections to the total possible connections, is calculated as 0.036. Furthermore, the network displays a heterogeneity value of 1.079, indicating the degree of variation in the number of connections among nodes. Notably, the presence of 100 connected components suggests the existence of multiple subnetworks within the overall network structure.

**Figure 3:**
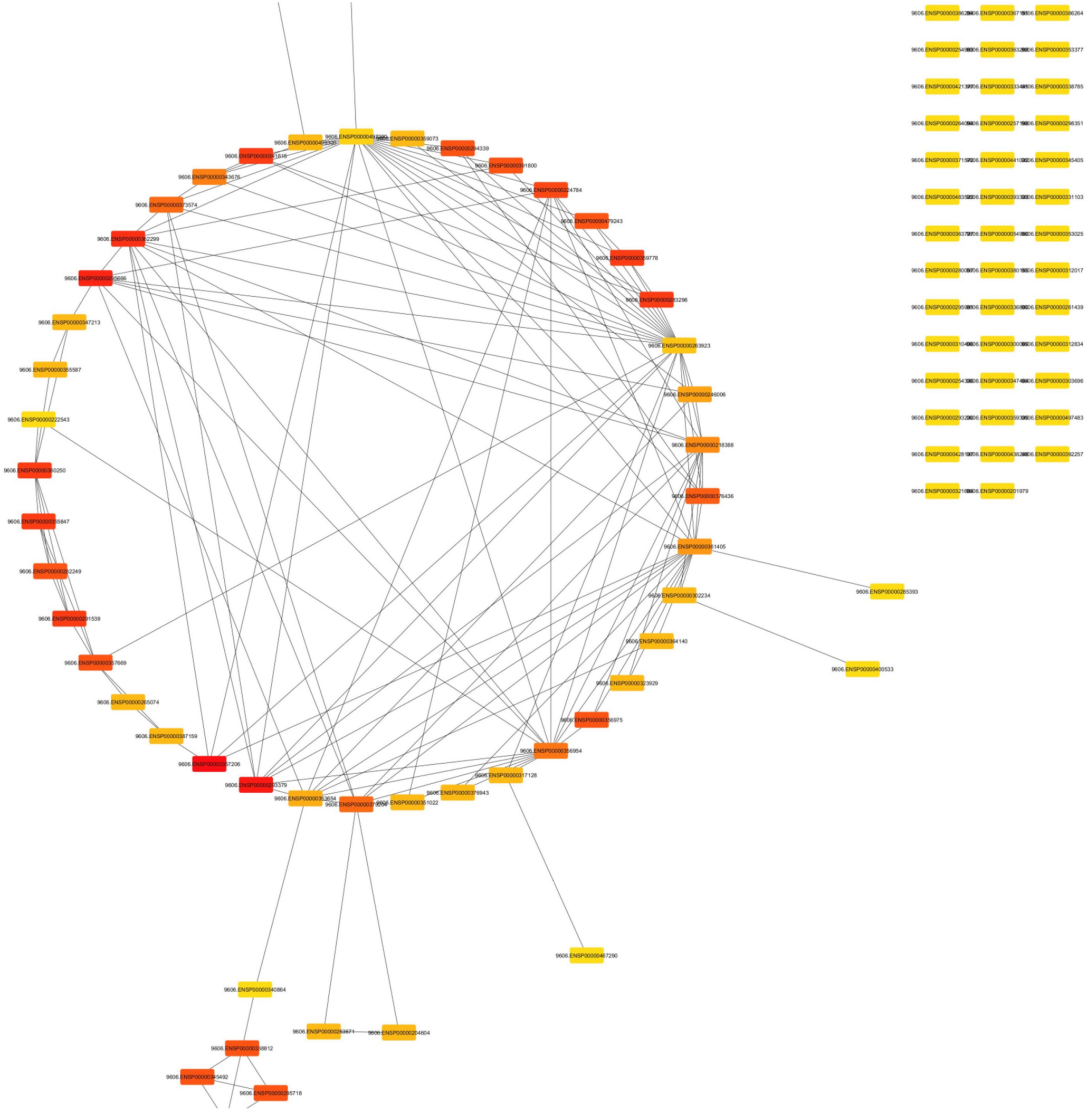
Interactome of the top 100 hub genes using CytoHubba.

Figure 3 showcases the interactome generated from analyzing the top 100 hub genes utilizing CytoHubba. These hub genes play crucial roles in biological networks and are central to various cellular processes. Among the top five hub genes highlighted in this interactome are IL17RC, CCN2, BMP7, TPM1, and TIMP1, all of which have been implicated in periodontal disease in conjunction with peri-implant disease. By examining this interactome, researchers can gain insights into the molecular interactions and pathways underlying the pathogenesis of these conditions. The figure offers a comprehensive visual representation of the key hub genes and their potential interactions. It facilitates further exploration and understanding of the molecular mechanisms involved in periodontal disease with peri-implant disease.

Table 1 summarizes the accuracy and class accuracy metrics derived from the application of gradient boosting in predicting interactome hub genes. The accuracy of 97% indicates the overall effectiveness of the algorithm in making correct predictions. In comparison, % class accuracy of 88% highlights the accuracy achieved for individual classes or categories within the dataset. These metrics are crucial indicators of the algorithm's performance and ability to identify hub genes involved in biological networks accurately.

**Table 1:** Accuracy and class accuracy of gradient boosting in predicting interactomic hub genes.

Model	AUC	CA	F1	Precision	Recall	LogLoss	Specificity
Gradient boosting	0.976	0.882	0.857	0.897	0.882	0.586	0.451

The Gradient Boosting model demonstrates robust performance in predicting interactomic hub genes associated with peri-implantitis and periodontitis. It discriminates between positive and negative samples, yielding an impressive Area Under the Curve (AUC) of 0.976. Moreover, the model achieves a high Classification Accuracy (CA) of 0.882, indicating that 88.2% of samples are accurately classified. The F1 score, indicative of the balance between precision and recall, stands at 0.857, signifying a satisfactory trade-off between the two metrics. Notably, the model exhibits a precision of 0.897, accurately identifying positive interactomic hub genes 89.7% of the time. Furthermore, it achieves a recall of 0.882, correctly identifying 88.2% of the positive interactomic hub genes. The model's capability extends to providing reliable probability estimates and effectively distinguishing between positive and negative samples, as evidenced by Table 1.

Table 2 comprehensively summarizes the confusion matrix results derived from network analysis based on hub and non-hub classifications. This matrix delineates the accuracy of classifying nodes into hub and non-hub categories. Notably, the table showcases the accuracy of correctly classifying hubs as hubs, achieving a perfect accuracy rate of 100%. Additionally, it highlights the accuracy of correctly identifying non-hubs as non-hubs, yielding a rate of 87.5%. However, the table also reveals the challenge in accurately classifying non-hubs, with only a 12.5% accuracy rate for non-hubs incorrectly classified as non-hubs. These results offer valuable insights into the performance of hub and non-hub classifications in network analysis, aiding in evaluating and refining classification algorithms.

**Table 2:** Confusion matrix results for network analysis based on hub and non-hub classifications.

	Predicted		
Actual	Hub	100.0%	12.5%
	Non-hub	0.0%	87.5%

**Figure 4:**
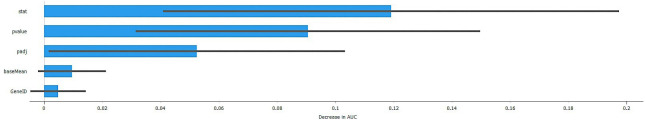
SHAP plot of the gradient boosting model.

A SHAP plot serves as a visual depiction of machine learning model predictions employing the SHAP (Shapley Additive explanations) value method. It elucidates the contribution of each feature to the prediction through vertical bars, where longer bars denote a more substantial impact, and positive bars signify an enhancement in prediction. The plot commences with a base value and iteratively incorporates or deducts each feature's contribution to ascertain the final prediction (Figure 4).

**Figure 5:**
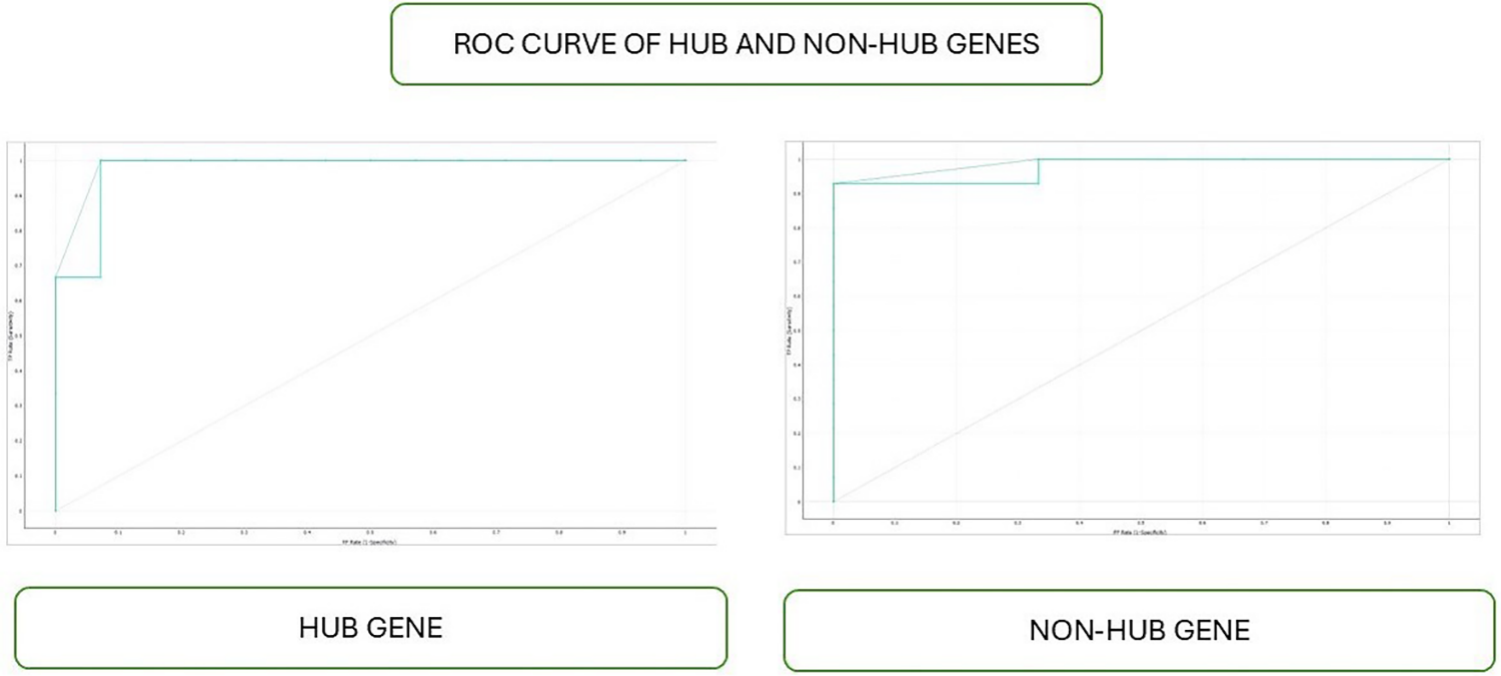
ROC curve of hub and non-hub genes.

The Receiver Operating Characteristic (ROC) curve serves as a graphical representation of the performance of a binary classification model. It is constructed by plotting the True Positive Rate (TPR), also known as sensitivity, against the False Positive Rate (FPR) at various classification thresholds. TPR represents the proportion of correctly predicted positive instances out of all positive ones. In contrast, FPR represents the proportion of incorrectly predicted positive instances out of all negative instances (Figure 5).

**Figure 6:**
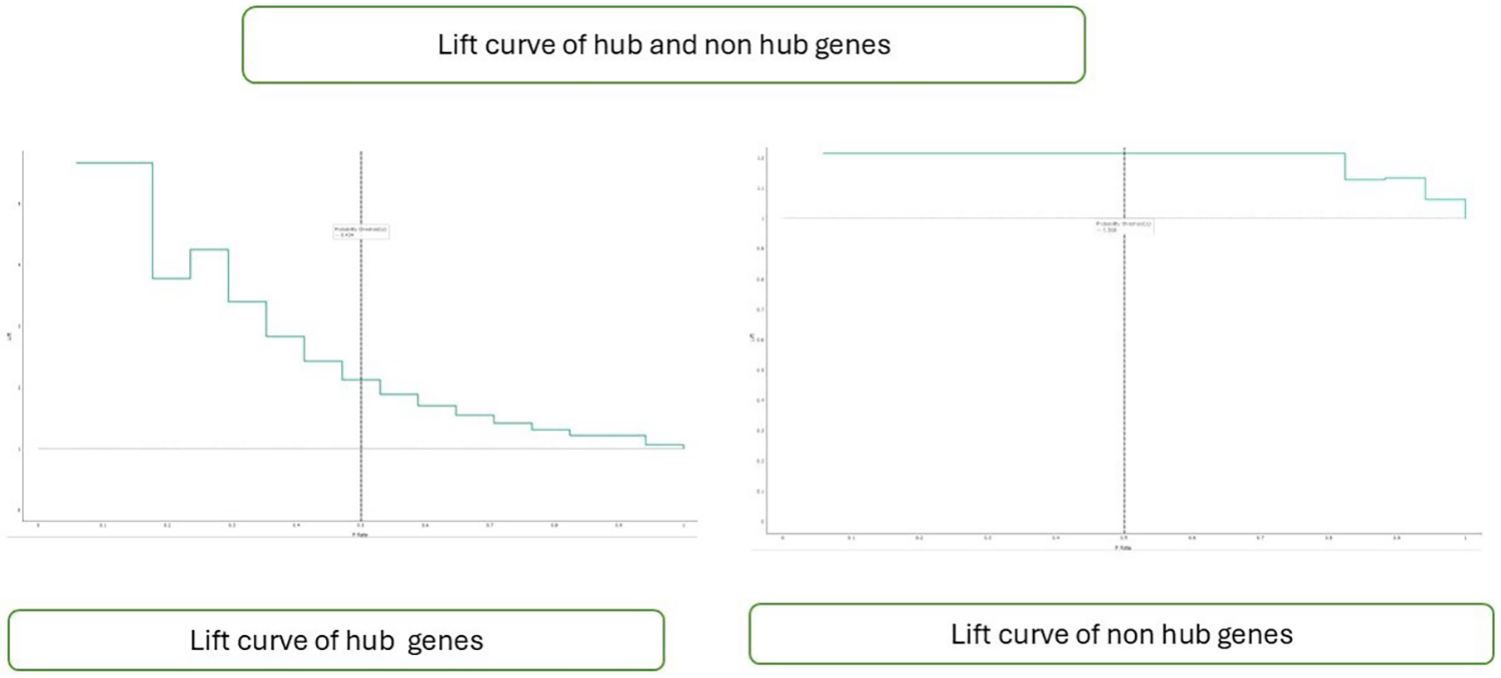
Lift curve of hub and non-hub genes.

Figure 6 presents the Lift curve depicting the effectiveness of a binary classification model in identifying positive instances, specifically for both hub and non-hub genes. This graphical representation is particularly valuable in scenarios where positive instances are rare or of paramount importance to predict accurately. The Lift curve is constructed by plotting the proportion of positive instances captured on the *y*-axis against the proportion of the dataset examined on the *x*-axis. A higher Lift curve indicates superior performance compared to random selection. This curve aids in evaluating the model's ability to prioritize positive instances. It provides insights into its practical utility in genomic research and other applications where accurate identification of rare positive instances is essential.

**Figure 7:**
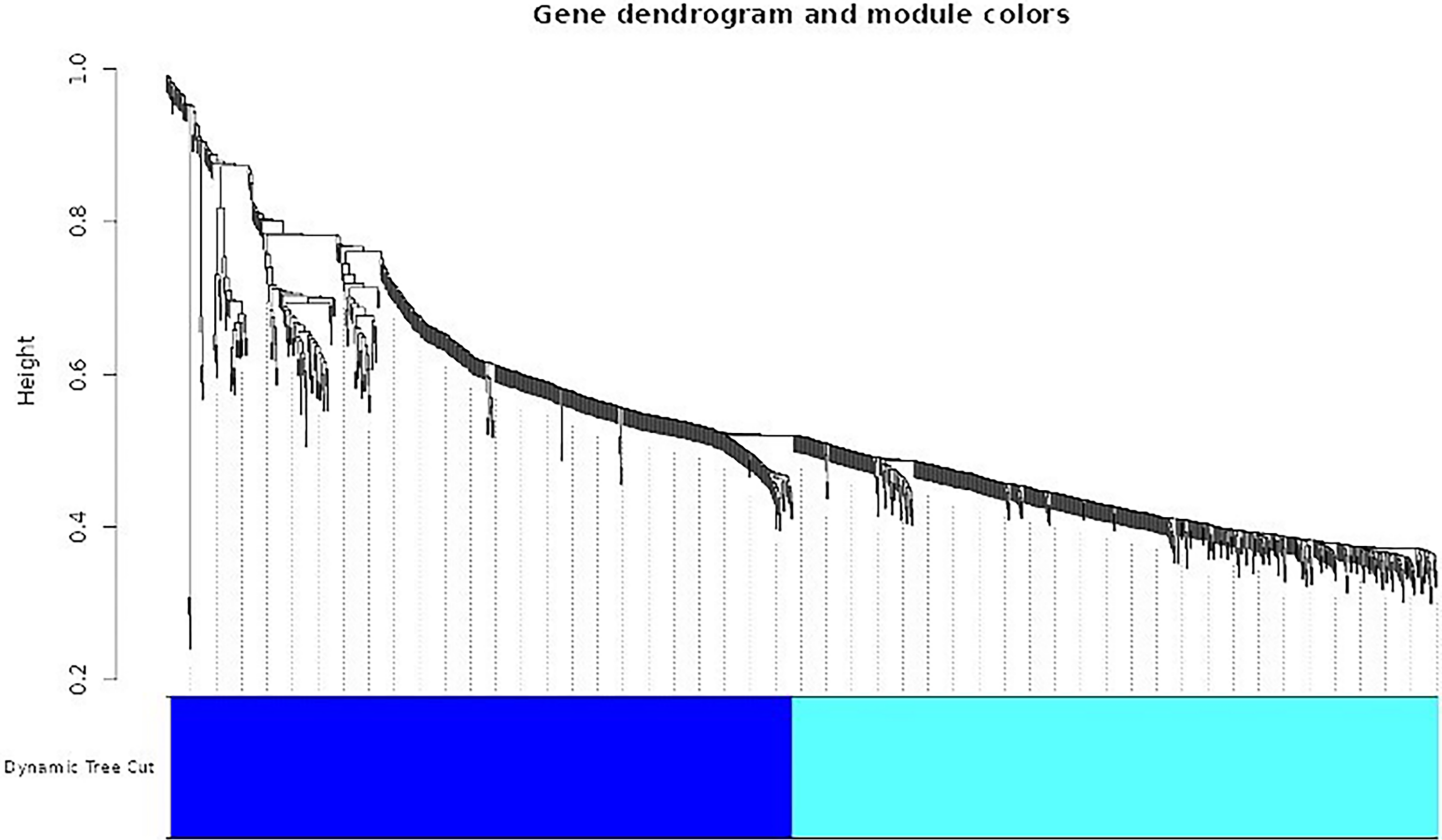
Dendrogram of top modules.

Figure 7 presents a dendrogram showcasing the top modules identified through dissimilarity clustering. Modules are groups of tightly interconnected genes sharing similar expression patterns. This dendrogram visualizes modules in different colors, with colored lines indicating individual genes' memberships to specific modules.

The WGCNA software facilitated the construction of a co-expression network utilizing 1,000 differentially expressed genes. Hierarchical clustering and a topological overlap matrix were generated for analysis to ensure a scale-free topology, employing a soft thresholding power 10. The figure displays a dendrogram illustrating the top analytical modules identified through dissimilarity clustering. Each module is represented by a distinct color in the dendrogram, with genes within each module closely linked and marked by colored lines. This method effectively identifies modules comprising densely coupled genes, unveiling gene regulatory networks and co-regulated biological processes.

**Figure 8:**
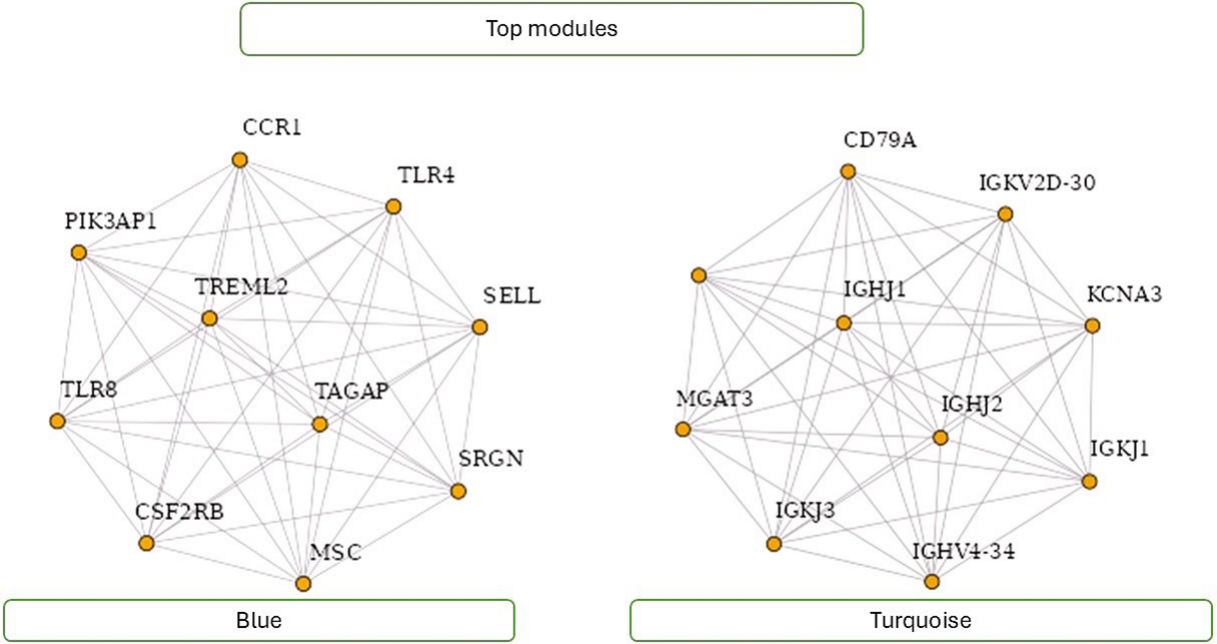
Top modules with ten hub genes.

Figure 8 showcases the top modules identified through WGCNA analysis. The figure highlights ten hub genes within these modules, indicating their pivotal role in mediating interactions and regulating biological processes. Researchers can gain valuable insights into the underlying molecular mechanisms and regulatory networks associated with specific phenotypes or conditions by focusing on these top modules and hub genes. Figure 8 visualizes key gene modules and hub genes identified through WGCNA analysis, facilitating further exploration and understanding of complex biological systems.

**Figure 9:**
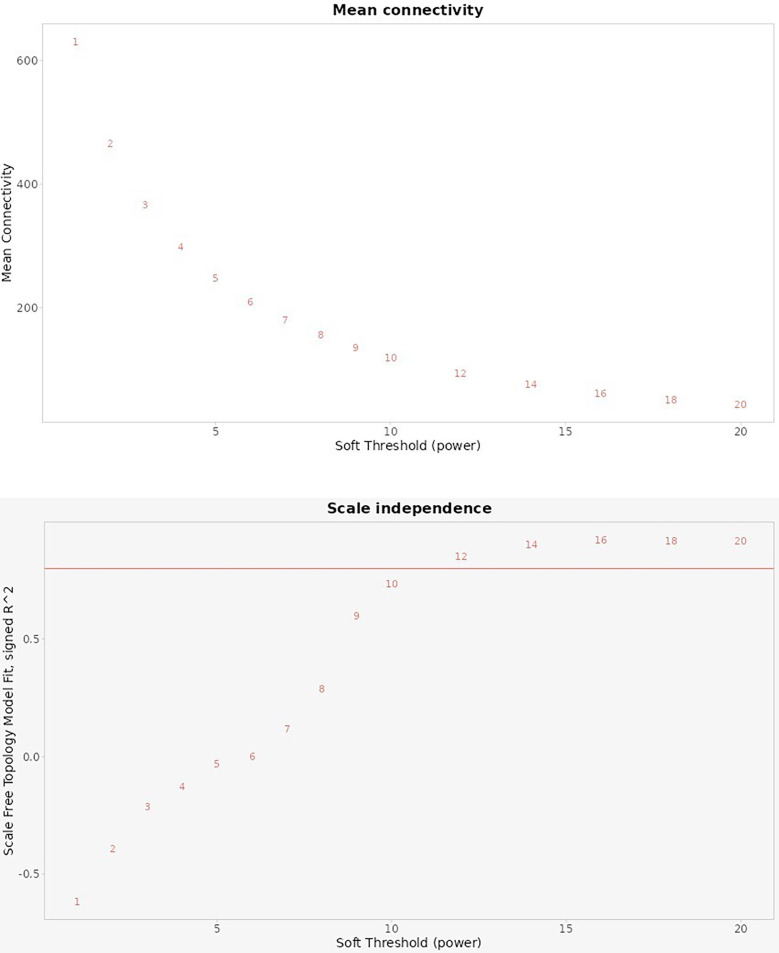
Analysis of scale-free topology fitting index *R*^2^ and mean connectivity.

Figure 9 illustrates the selection of an optimal soft-thresholding power in the weighted gene co-expression network analysis, using two key metrics: Mean Connectivity and Scale Independence.

Top Plot (Mean Connectivity): This chart shows the average connectivity of nodes (genes) within the network across different soft threshold powers. As the soft threshold power increases, the mean connectivity decreases, reflecting a reduction in the number of connections each gene has. This trend suggests that increasing the power enhances the specificity of connections, focusing on stronger correlations and reducing weaker, potentially noisy connections.

Bottom Plot (Scale Independence): This chart displays the scale-free topology fitting index *R*^2^ across various soft threshold powers. The goal in WGCNA is to select a power that achieves a high *R*^2^ value (typically above 0.8), indicating that the network approximates a scale-free topology. In this case, the red line indicates an *R*^2^ threshold of 0.8. As the soft threshold power increases, the *R*^2^ values approach or exceed 0.8 around a specific power level, indicating the network's suitability for scale-free topology at that point.

Together, these plots help determine the optimal power to achieve a balance between preserving key gene connections (higher connectivity) and achieving a scale-free network structure (high *R*^2^ value). This balance is essential in identifying meaningful gene modules that correlate with biological traits in the study of peri-implantitis and periodontitis.

**Figure 10:**
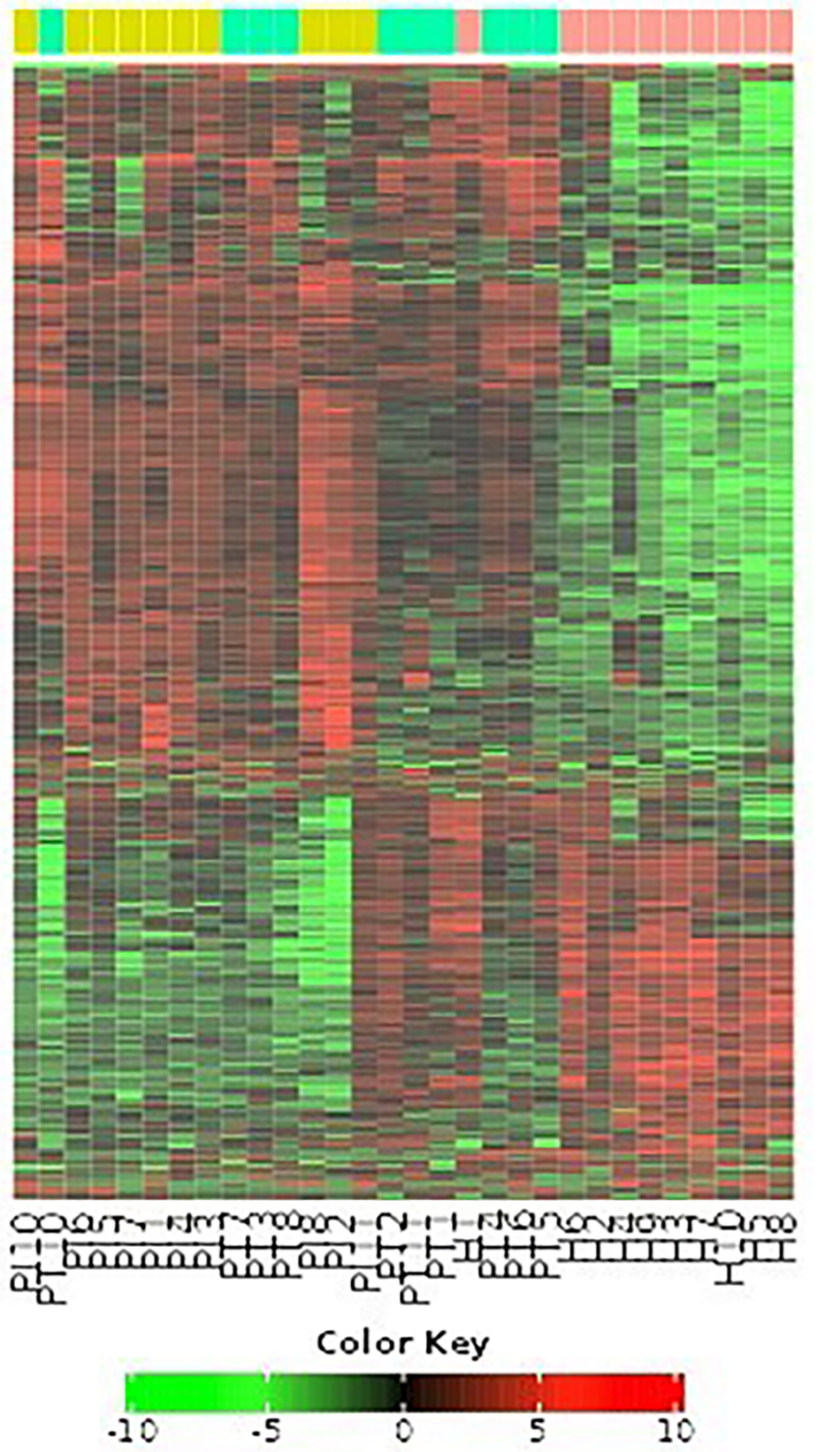
Heat map and topological overlap matrix of analyzed genes.

Figure 10 comprehensively visualizes the analyzed genes through a heat map graphic and a topological overlap matrix. The heat map provides a graphical representation of the pairwise similarities between genes, allowing for the identification of clusters or modules of co-expressed genes. Brighter colors in the heat map indicate stronger similarities, while darker colors signify weaker similarities.

Table 3 provides an overview of the enriched genes associated with module hub genes, categorized into clusters based on highly enriched and statistically significant pathways. Each pathway listed in the table is accompanied by the number of genes enriched within that pathway, ranging from 53 to 95. Noteworthy pathways identified include defense response and external biotic stimulus-response, highlighting the biological processes and mechanisms that regulate gene expression within these modules.

**Table 3:** Enriched genes of module hub genes.

	FDR	nGenes	Pathway
1	2.62E-16	95	Defense response
2	8.24E-16	84	Response to external biotic stimulus
3	8.24E-16	90	The biological process involves interspecies interaction between organisms.
4	8.24E-16	84	Response to other organism
5	2.25E-15	53	Response to bacterium

Table 4 presents an overview of enriched module hub genes grouped into clusters, highlighting pathways associated with diverse biological processes. The table categorizes these genes based on their involvement in pathways, with each cluster representing a distinct functional module enriched with hub genes. Pathways listed in Table 4 range from 53 to 95 genes, reflecting the complexity and diversity of biological processes regulated by these hub genes.

**Table 4:** Enriched module hub genes in clusters.

	FDR	genes	Fold enriched	Pathway
1	1.05E-11	33	4.667488341	Cytokine-cytokine receptor interaction
2	4.82E-10	19	7.405813953	Rheumatoid arthritis
3	1.65E-08	17	6.865757768	IL-17 signaling pathway
4	3.28E-08	17	6.475657895	Viral protein interaction with cytokine and cytokine receptor

The analysis results reveal the involvement of cytokine-cytokine receptor interaction, rheumatoid arthritis, IL-17 signaling pathway, and viral protein interaction with cytokine and cytokine receptors. These pathways play pivotal roles in various biological processes, offering valuable insights into the potential functions of the analyzed genes.

Table 5 provides an overview of the cellular components associated with hub genes demonstrating high connectivity within a biological network. Cellular components represent subcellular structures or compartments where proteins, encoded by hub genes, are predominantly localized and exert their functional roles.

**Table 5:** Cellular components of hub genes.

	FDR	nGenes	Pathway size	Fold enriched	Pathway
1	3.14E-33	199	4,673	2.198723934	Extracellular region
2	2.31E-19	150	3,577	2.075214341	Extracellular space
3	5.24E-13	62	987	2.994557823	Secretory granule
4	8.82E-12	66	1,165	2.683848797	Secretory vesicle
5	7.73E-11	44	603	3.373650108	External encapsulating structure

## Discussion

Dental implants are a popular treatment for tooth loss, offering functional, aesthetic, and quality-of-life benefits. However, placing implants in periodontitis-prone patients can be challenging (9, 26). Factors such as patient compliance and peri-implant microbiota significantly affect implant success. Previous studies highlight the high prevalence of peri-implant disease in patients with advanced periodontitis, underscoring the need for regular maintenance and monitoring. These studies emphasize the correlation between implants and the likelihood of disease, stressing the importance of oral hygiene, smoking cessation, plaque control, and supportive care (4–6).

Previous research examined plaque samples from individuals with teeth or implants to explore oral microbiome dysbiosis in periodontitis and peri-implantitis. Results indicated that inflammation reduced subgingival connectivity and increased supragingival connectivity (27, 28). Subgingival microbiota stability decreased with periodontitis and peri-implantitis. The findings highlight that hub species are important in future research because dysbiosis affects bacterial correlations, community architecture, and local stability under these conditions (13).

Previous studies found that peri-implantitis lesions had more pronounced inflammatory cell infiltrates and a larger inflammatory cell infiltrate extending to the bone crest than periodontitis (1, 11–13). Another study (7) compared the histologic features of severe periodontitis and severe peri-implantitis lesions, revealing larger and more advanced peri-implantitis lesions with distinct onset and progression mechanisms. This study also identified differential gene expression using the GEOR TOOL (27) (Figures 1–3).

The dendrogram displays modules in different colors, revealing gene regulatory networks and co-regulated biological processes using WGCNA analysis. The study utilized a hierarchical clustering tree and topological overlap matrix, revealing a scale-free topology fitting index *R*^2^ and mean connectivity for various soft threshold powers, with an *R*^2^ value of 0.9 identifying two modules of gene co-expression (Figures 7–10; Tables 3–5). Top genes identified through ontology analysis reveal cytokine receptors and IL-17 pathways, commonly involved in periodontal and peri-implant disease.

Top hub genes include IL17RC, CCN2, BMP7, TPM1, and TIMP1, which are involved in periodontal and peri-implant diseases. Studies involving hub genes include the IL-23/IL-17 axis (29), crucial for periodontitis development, promoting proinflammatory cytokines and alveolar bone loss. Another study indicates a reverse relationship between IL-23R and IL-17RA in chronic and aggressive periodontitis patients, potentially linked to RANKL activation and alveolar bone loss. Previous studies explore the role of IL-17 in peri-implantitis and periodontal diseases, revealing a correlation between IL-17 genotypes and susceptibility, suggesting a molecular-level control of IL-17 release. Elevated IL-17 levels are observed in periodontitis cases. CCN2 (30), a CCN protein, is crucial for bone and cartilage growth, orofacial development, mandibular morphogenesis, tooth germ development, and tissue remodeling linked to fibrotic disorders and periodontal fibrosis. CCN2/CTGF (31) is essential for periodontal tissue development and regeneration, with stronger expression in sparse cell cultures and enhanced by TGF-β. It stimulates DNA synthesis, cell growth, and specific marker expression. ACTN1 and ACTN2 proteins, known for crosslinking F-actin and anchoring actin to intracellular structures, were associated with FAM49B and TPM1 in mitochondria and the cytoskeleton in periodontal and peri-implant diseases. TIMPs, an MMP-9 inhibitor (32), may be biomarkers for diagnosing and monitoring periodontitis by reducing MMP activity linked to tissue destruction and disease progression.

The accuracy and class accuracy of gradient boosting showed 97% and 88%, respectively, in predicting interactome hub genes (Tables 1, 2; Figures 4–6). The gradient boosting approach for predicting interactome hub genes requires validation with independent datasets to assess its reliability and generalizability. Integrating multi-omics data can comprehensively understand the regulatory mechanisms underlying hub gene selection biology of hub gene selection in periodontal diseases with peri-implantitis. This study uses a network-based approach to classify interactome hub genes associated with periimplantitis and periodontitis, revealing key genes involved in inflammation, osteoclast genesis, angiogenesis, and immune response regulation. The study identified 216 genes, with top hub genes like IL17RC, CCN2, BMP7, TPM1, and TIMP1 involved in periodontal disease. The study identifies key genes for developing novel treatment strategies and highlighting molecular pathways. However, validation through independent datasets is crucial for confirming its reliability. Integrating multi-omics data could provide a holistic view of biological processes, leading to more nuanced insights into the pathogenesis of periodontal diseases. Data quality and availability issues, as well as inherent complexity of machine learning models, pose challenges. As the dataset was sourced from the NCBI GEO database, the sample size was predetermined by the original study authors, and we are thus limited by the data available within this resource. While our analysis is constrained by this existing sample size, we acknowledge the importance of validating our findings with larger datasets. Future research should address these limitations, incorporate advanced methods for interpreting machine learning outputs, and explore the functional roles of predicted hub genes through laboratory experiments.

## Conclusions

Gradient boosting is a promising method for predicting interactome hub genes, revealing key regulatory genes in biological networks associated with peri-implantitis and periodontitis. However, independent and multi-omics data validation is needed to improve model robustness and generalizability. Experimental validation is also crucial to confirm the functional relevance of these predicted genes. Furthermore, understanding the regulatory mechanisms of these hub genes can lead to the development of targeted therapies and improved diagnostic tools for periodontal and peri-implant diseases. Future research should focus on integrating advanced computational techniques with experimental approaches to enhance the precision and applicability of hub gene predictions in clinical settings.

## Data Availability

The raw data supporting the conclusions of this article will be made available by the authors, without undue reservation.
